# Precision and type I error rate in the presence of genotype errors and missing parental data: a comparison between the original transmission disequilibrium test (TDT) and TDTae statistics

**DOI:** 10.1186/1471-2156-6-S1-S150

**Published:** 2005-12-30

**Authors:** Sandra Barral, Chad Haynes, Mark A Levenstien, Derek Gordon

**Affiliations:** 1 Laboratory of Statistical Genetics, Rockefeller University, New York, NY 10021 USA

## Abstract

**Background:**

Two factors impacting robustness of the original transmission disequilibrium test (TDT) are: i) missing parental genotypes and ii) undetected genotype errors. While it is known that independently these factors can inflate false-positive rates for the original TDT, no study has considered either the joint impact of these factors on false-positive rates or the precision score of TDT statistics regarding these factors. By precision score, we mean the absolute difference between disease gene position and the position of markers whose TDT statistic exceeds some threshold.

**Methods:**

We apply our transmission disequilibrium test allowing for errors (TDTae) and the original TDT to phenotype and modified single-nucleotide polymorphism genotype simulation data from Genetic Analysis Workshop. We modify genotype data by randomly introducing genotype errors and removing a percentage of parental genotype data. We compute empirical distributions of each statistic's precision score for a chromosome harboring a simulated disease locus. We also consider inflation in type I error by studying markers on a chromosome harboring no disease locus.

**Results:**

The TDTae shows median precision scores of approximately 13 cM, 2 cM, 0 cM, and 0 cM at the 5%, 1%, 0.1%, and 0.01% significance levels, respectively. By contrast, the original TDT shows median precision scores of approximately 23 cM, 21 cM, 15 cM, and 7 cM at the corresponding significance levels, respectively. For null chromosomes, the original TDT falsely rejects the null hypothesis for 28.8%, 14.8%, 5.4%, and 1.7% at the 5%, 1%, 0.1% and 0.01%, significance levels, respectively, while TDTae maintains the correct false-positive rate.

**Conclusion:**

Because missing parental genotypes and undetected genotype errors are unknown to the investigator, but are expected to be increasingly prevalent in multilocus datasets, we strongly recommend TDTae methods as a standard procedure, particularly where stricter significance levels are required.

## Background

One of the most-widely used family-based linkage tests in the presence of association is the original transmission disequilibrium test (TDT) statistic [1]. There are two principal limitations regarding the robustness of this original statistic: i) missing parental genotype data; and ii) undetected genotyping errors.

It has been shown [2-4] that both factors may cause an increase in the type I error rate of the statistic, thereby inflating the false-positive rate among the reported linkages. However, no studies to date have quantified the impact that both factors jointly have on inflation of type I error for the original TDT statistic. We designed the TDTae statistic [4,5] to address these factors. The TDTae is a likelihood ratio test of linkage in the presence of association for general pedigrees. Simulation studies [5] suggest that the TDTae statistic is robust (in terms of maintaining correct type I error) to the presence of these factors. The TDTae maximizes the likelihood of the data over the genotypic relative risk parameters (R_1_, R_2_), population genotype frequencies, and error model parameters under the null hypothesis that the genotypic relative risks are equal to 1 (R_1 _= R_2 _= 1) and under the alternative hypothesis that at least one of the genotypic relative risks is not equal to 1 (R_1 _≠ 1 or R_2 _≠ 1).

The genotype relative risks for a di-allelic locus with wild-type allele + and disease allele d are defined as:

R_1 _= Pr(affected|+d)/ Pr(affected|++)

R_2 _= Pr(affected|dd)/ Pr(affected|++).

It should be noted that when a multiplicative mode of inheritance is assumed, (R_2 _= R_1_^2^), the TDTae statistic reduces to the original TDT statistic [6,7].

An important, but overlooked, question concerns the robustness of the precision of TDT statistics in the presence of missing parental genotype data and undetected genotyping error. From this point forward, we shall define precision score as the absolute distance between the location of the disease gene and that marker whose TDT statistic is significant at some pre-specified significance level threshold. While this question has been addressed previously in linkage studies [8,9] (for factors such as sibling relative risk and locus heterogeneity), it has not, to date, been considered for TDT statistics, particularly in the presence of the two above-mentioned factors.

## Methods

The datasets we considered for all analyses were the simulated trait data sets from the Genetic Analysis Workshop 14 (GAW14) Workshop. We defined as affected those individuals who were affected (in the phenotypic data file, column referring to affection status of 2 = affected) and for whom phenotype E is present (in the phenotypic data file phenotype E = 1, meaning that the trait is present).

We considered only the three subpopulations consisting of nuclear families. That is, we excluded the New York pedigrees from our analyses, because our TDTae method required significantly more computational time to perform analysis of even one replicate of the New York data. We had knowledge of the true model at the time of the analysis.

### Genotype data modification

All replicates were modified by randomly removing 10% of the parental genotypes and also introducing errors to the remaining genotypes using the Sobel-Papp-Lange (SPL) error model [10] with the penetrances specified in Table 1. In this table, the coded genotype 1 refers to the 11 genotype, the coded genotype 2 refers to the heterozygote 12, and the coded genotype 3 refers to the 22 genotype. Using this table, we observe, for example, that the homozygote 11 had a 1% probability of being misclassified as a 12 and vice versa.

### Test statistics

**Figure 1:**
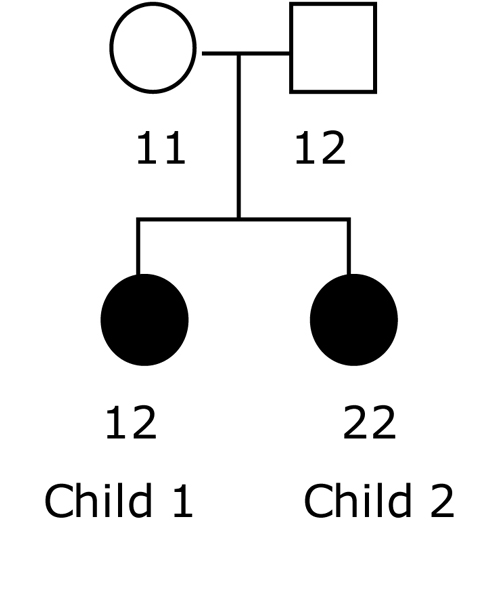
**Example of family analyzed by TDT and TDTae statistics**. In our analysis, the original TDT statistic only used the trio consisting of the parents and child-1 because the trio with the parents and child-2 shows a Mendelian inconsistency. The TDTae was computed for both trios.

We performed both type I error and precision score analyses by computing the original TDT and the TDTae statistics. The basic unit considered for the analysis within the nuclear families was a trio with two genotyped parents and at least one affected child. For the original TDT [1], those trios with only one genotyped parent were ignored. Also, the original TDT statistic only analyzed those trios that showed Mendelian consistency for a given marker, while the TDTae statistic analysed all trios with an affected child. An example of a family analyzed by each statistic is provided in Figure 1.

After computing both statistics for all markers in each of the 100 replicates for either the type I error or precision score study, we selected the subset of markers over all the replicates with *p*-values less than 0.05 (5%), 0.01 (1%), 0.001 (0.1%), and 0.0001 (0.01%).

### Empirical type I error rate

We modified the simulated dataset of 95 single-nucleotide polymorphisms (SNPs) markers from chromosome 7, which does not harbor any disease locus, as specified above (Genotype data modification) to compute the empirical type I error rate in the presence of the aforementioned factors. We defined the empirical type I error rate for each statistic (TDT or TDTae) at a given threshold (5%, 1%, 0.1%, and 0.01%) as the proportion of SNPs from the total of 9,500 markers over all 100 replicates that showed *p*-values less than 0.05, 0.01, 0.001, or 0.0001, respectively.

### Precision score study

**Figure 2:**
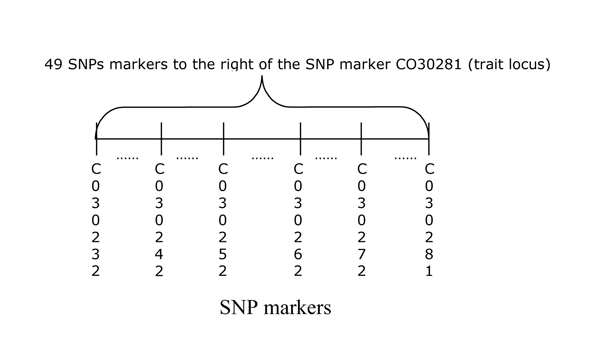
Map of the SNPs markers on chromosome 3 used in the analysis.

For the precision score study we considered the SNP trait locus at the end of chromosome 3 and 49 SNPs to one side of it (an average intermaker distance of 3 cM; see Figure 2). As above, we modified the genotype data (Genotype data modification) and analyzed the 100 replicates, each one consisting of 50 SNPs markers on chromosome 3 for all nuclear families across the three subpopulations.

To determine the precision score, we first computed the distance (in marker units) from the trait locus to a marker that showed a significant *p*-value for a given statistic at a given threshold significance level. The distance is the absolute difference between the trait locus position and marker's position. For example, if marker 35 in a replicate had a *p*-value less than the threshold for a given statistic, its distance to the trait locus is |50 - 35| = 15 and therefore it precision score for that significance level is 15.

We computed the distribution of the precision score for all significance levels with each statistic by considering various percentiles (minimum, first quartile, median, third quartile and maximum).

## Results

### Empirical type I error rates

Table 2 shows the results of the empirical type I error rates for each statistic. The TDT and TDTae columns report the proportion of replicates in which the *p*-values were less than the value x/100. The values reported in parentheses are the lower and upper end points of the 95% confidence intervals computed using the method implemented in the BINOM program .

Table 2 shows that the original TDT has appreciable inflation in type I error rates at all significance levels. Furthermore, the inflation increases as the significance level decreases. For example, there is an approximate 6-fold increase in the type I error at the 5% significance level (28.8/5), while there is a 170-fold increase in type I error at the 0.01% significance level (0.017/0.0001).

It is important to note that this inflation is for data with relatively small genotype error rates. We suspect that there is a compounding effect of the type I error inflation for the original TDT when a dataset contains both genotype errors and missing parental genotypes.

### Precision score study

Based on the median results for each distribution (Table 2; 1% significance level), half of the significant markers for the original TDT were located at a distance of at least 23, 21, 15, or 7 units from the trait locus at the 5%, 1%, 0.1%, and 0.01% significance levels, respectively. By contrast, at least half of the significant markers for the TDTae were at distance no more than 13, 2, 0, or 0 units from the trait locus.

## Discussion

The results of our analysis on the simulated data suggest that when the alternative hypothesis is true, the TDTae statistic may be a more precise indicator of the trait location than the original TDT statistic in the presence of missing parental data and genotyping errors.

Regarding the empirical type I error rate, we have shown that the TDTae statistic is able to maintain proper type I error rate in the presence of errors for these simulated datasets. The original TDT statistic shows a highly inflated false-positive rate when there are missing parental genotypes and random genotyping errors in the dataset. The results of our simulations suggest that the inflation in type I error increases as the significance level becomes more stringent.

These results have significant consequences for TDT analyses with large numbers of markers, for example studies using microarray technologies [11]. Many of the genotype errors will not be detected [12], potentially inflating type I error for the original TDT statistic. Because: i) more stringent significance levels are needed to correct for the multiple testing issue; and ii) we observe (Table 2) that type I error inflation is more severe as the significance level becomes more stringent, we strongly recommend that researchers performing TDT analyses on large numbers of markers use methods [13,14] like the TDTae that incorporate genotype errors into the analysis. Software for our method is available at: .

## Conclusion

We strongly recommend that researchers apply TDTae methods when missing parental genotypes or undetected genotype errors are present and when the number of markers is large. We reason that when more markers are tested, more stringent significance levels are required to correct for multiple testing issues. However, the false-positive rate increases disproportionately for original TDT methods as the significance level becomes more stringent, while our work here suggests that the TDTae maintains proper type I error rates in the presence of missing parental genotype data and genotype errors, even for more stringent significance levels.

## Abbreviations

GAW14: Genetic Analysis Workshop 14

SLP: Sobel-Papp-Lang

SNP: Single-nucleotide polymorphism

TDT: Transmission disequilibrium

TDTae: Transmission disequilibrium test allowing for errors

## Authors' contributions

SB performed all statistical analyses and wrote the majority of the manuscript. CH developed the computer programs to introduce the errors and remove parental genotype data. MAL developed the ideas for the precision study and wrote a portion of the Results section. DG proposed the research for the GAW14 dataset, supervised all the research, and reviewed all versions of the manuscript for scientific content and grammar.

## Figures and Tables

**Table 1:** Sobel-Papp-Lange error model penetrances values

True coded genotype	Observed coded genotype		
	
	1	2	3
1	0.989	0.01	0.001
2	0.01	0.98	0.01
3	0.001	0.01	0.989

**Table 2:** Empirical significance levels and 95% confidence intervals for original TDT and TDTae

Significance level	TDT (95% CI)	TDTae (95% CI)
5%	0.288 (0.279–0.297)	0.052 (0.047–0.057)
1%	0.148 (0.141–0.155)	0.012 (0.009–0.014)
0.1%	0.054 (0.049–0.059)	5.1 × 10^-4 ^(1.4 × 10^-4^-1.3 × 10^-3^)
0.01%	0.017 (0.015–0.020)	1.3 × 10^-4 ^(3.3 × 10^-6^-7.2 × 10^-4^)

**Table 3:** Distribution of the precision scores for significant markers for original TDT and TDTae at various significance levels.

	5% level		1% level		0.1% level		0.01% level	
				
	Original TDT	TDTae	Original TDT	TDTae	Original TDT	TDTae	Original TDT	TDTae
Minimum	0	0	0	0	0	0	0	0
10 Percentile	2	0	0	0	0	0	0	0
1^st ^Quartile	10	1.5	8.5	0	2	0	0	0
Median	23	13	21	2	15	0	7	0
3^rd ^Quartile	39	34	38	20	37	0	22	0
90^th ^Percentile	45	44	45	40	45	9.6	43	0
Maximum	49	49	49	48	49	34	49	24
